# Graphene Oxide-Reinforced Alginate Hydrogel for Controlled Release of Local Anesthetics: Synthesis, Characterization, and Release Studies

**DOI:** 10.3390/gels8040246

**Published:** 2022-04-16

**Authors:** Cuong Hung Luu, Giang Nguyen, Thanh-Tuyen Le, Thanh-Mai Ngoc Nguyen, V. H. Giang Phan, Mohanapriya Murugesan, Ramya Mathiyalagan, Lu Jing, Gopinathan Janarthanan, Deok Chun Yang, Yi Li, Thavasyappan Thambi

**Affiliations:** 1 Biomaterials and Nanotechnology Research Group, Faculty of Applied Sciences, Ton Duc Thang University, Ho Chi Minh City 70000, Vietnam; luuhungcuong145@gmail.com (C.H.L.); giangnguyen.8097@gmail.com (G.N.); tuyenle27@gmail.com (T.-T.L.); mainguyen2138@gmail.com (T.-M.N.N.); phanvuhoanggiang@tdtu.edu.vn (V.H.G.P.); 2 Graduate School of Biotechnology, College of Life Sciences, Kyung Hee University, Yongin si 17104, Korea; priyabioinfo@khu.ac.kr (M.M.); ramya@khu.ac.kr (R.M.); dcyang@khu.ac.kr (D.C.Y.); 3 Division of General Education, Seokyeong University, Seoul 02173, Korea; jinglu911@hotmail.com; 4 Research and Development Department, ECOWORLD Pharm Co., Ltd., Damyang-gun 57304, Korea; biotechgopi@gmail.com; 5 College of Materials and Textile Engineering, Nanotechnology Research Institute, Jiaxing University, Jiaxing 314001, China; 6 School of Chemical Engineering, Sungkyunkwan University, Suwon 16419, Korea

**Keywords:** lidocaine, graphene oxide, alginate, local anesthesia, injectable hydrogel

## Abstract

In pain relief, lidocaine has gained more attention as a local anesthetic. However, there are several side effects that limit the use of local anesthetics. Therefore, it is hypothesized that a hydrogel system with facile design can be used for prolonged release of lidocaine. In this study, we developed a formulation comprises of sodium alginate (SA) and graphene oxide (GO) to prolong the release of lidocaine. The gelation was induced by physically crosslinking the alginate with Ca^2+^ ions. The formation of blank SA and GO-reinforced SA hydrogels was investigated with different concentration of Ca^2+^ ions. The controlled release of lidocaine hydrochloride (LH) on both hydrogel systems was studied in PBS solution. The GO-reinforced SA hydrogels exhibited more sustained release than SA hydrogels without GO. In vitro biocompatibility test in L929 fibroblast cells confirmed the non-toxic property of hydrogels. Furthermore, to prove the *in-situ* gelation and biodegradability of hydrogels the hydrogels were injected on mice model and confirmed the stable gel formation. The hydrogels implanted onto the subcutaneous tissue of hydrogels retained over one week. These results indicate that LH-loaded GO-reinforced SA hydrogel can be a potential biomaterial for controlled release of local anesthetics.

## 1. Introduction

It is inevitable that patients who underwent operation suffer from chronic post-surgical pain. Such pain can result in local physical trauma and even emotional drowsiness, which can lead to dysfunctional behaviors in social life [1,2,3]. Therefore, there is the thriving need for preventing and relieving undesirable complications. Peripheral use of local anesthetics (lidocaine, bupivacaine, procaine, dibucaine, etc.) are commonly exploited for analgesia through a single administration [4,5]. These medicines initiate the excitation and conduction processes that consequently prohibit the transmission of nerve impulses, thereby numbing a specific administered site [6]. Lidocaine, which is usually used in the form of hydrochloride salt, is an amide based local anesthetic. Typical properties of lidocaine are low molecular weight and highly lipophilic [7]. These factors favorably influence local anesthetic activity, resulting in great penetration through the nerve membrane, high degree of protein binding, rapid onset and long action time [8,9]. Clinically, due to the ideal pK_a_ of 7.9 and aqueous solubility, the ionized form of lidocaine is preferred and become the most prevalent local anesthetics [10].

Although the safety of local anesthetics is higher than that of general anesthetics, its clinical application is greatly limited by various side effects. Some of those issues are neurotoxicity and sharp fluctuating levels of plasma-drug concentration [11,12]. Moreover, sustaining administration of local anesthetic is challenging to carry out, which leads to weak compliance [12,13]. In addition, lidocaine could trigger numerous adverse effects, such as vasodilation, serious arrhythmias, and depleted peripheral resistance [14,15,16]. Therefore, versatile drug delivery systems have been engineered to facilitate prolongation of lidocaine release locally at the treatment site and lessen toxicity. Hydrogels are of great concern in acute and chronic pain management owing to their biocompatibility and their ability to control encapsulated therapeutic agents [8,17,18,19,20,21,22,23,24,25]. Although there have been diverse polymer-based hydrogel systems were investigated, sodium alginate (SA) has been gaining an intense interest. SA is a linear polysaccharide derivative of alginic acid composed of (1–4)-linked β-D-mannuronic (M) and α-L-guluronic (G) acids. It possesses prominent characteristics, including high adsorption capacity, cytocompatibility, non-immunogenicity and natural abundance [13,26,27]. In addition, SA is identified by the abundant anionic carboxylate groups distributed along the backbone chain which can interact with positively charged groups of drugs to generate ionic complexes and control sustaining release [28,29,30]. In case of lidocaine delivery, SA is adopted as the most appropriate material. However, uncontrolled degradation properties of SA hydrogels induce the rapid lidocaine release and therefore requires frequent injection.

In order to improve the stability and control degradation of hydrogel network system, graphene oxide (GO) was introduced to the hydrogel network [31]. Apart from graphite, GO is a mono-layer and oxidized derivate of it, which is overexpressed with plentiful oxygen-enriched functionalities on graphene-based planar sheets, thus making it have high solubility in water as well as organic solvents [29,32]. Furthermore, the oxygenated groups, especially hydroxyls and carboxylic acids, can further formulate the ionic complexes with the basic groups of lidocaine hydrochloride (LH) [33,34]. Altogether, GO was considered as the best promising combination for any conventional hydrogel system. 

**Scheme 1:**
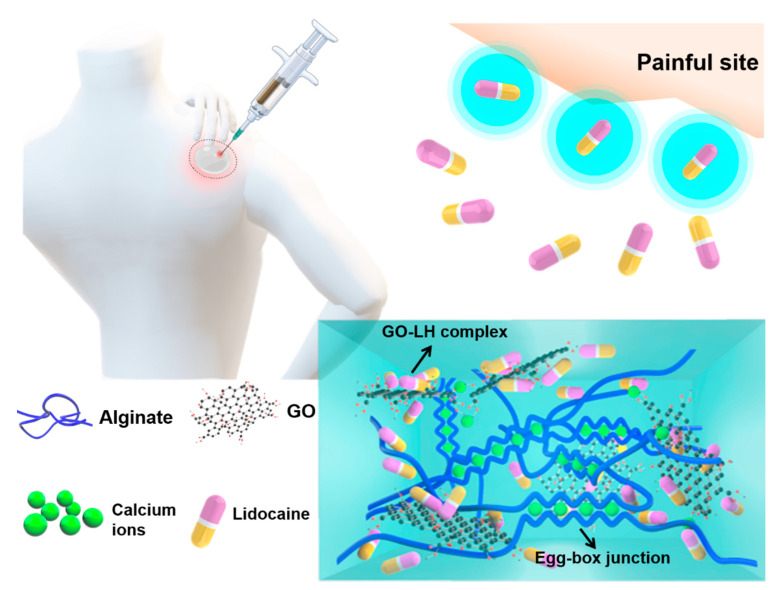
Illustrative models of GO-SA hydrogel physically crosslinked by divalent Ca^2+^ cations for LH delivery.

In this study, we developed injectable hydrogel system using GO-reinforced SA (GO-SA) hydrogel system for the controlled release of LH (Scheme 1). The harmonious association of LH with GO-SA hydrogels, verified by macroscopic observation and microscopic analysis. The physicochemical properties of hydrogels were assessed by using Fourier-transformed infrared spectroscopy (FT-IR), thermogravimetric analysis (TGA), ionic complexation and degradation studies, while the morphological and geometrical properties were analyzed by scanning electron microscopy (SEM). Drug loading capacity and release profile of LH were conducted to set a comparison between GO-SA and GO-free SA hydrogel systems in order to clarify the aid of GO in the drug interaction rate. In vitro biocompatibility of GO-SA and GO-free SA hydrogels were confirmed by co-culturing them with L929 fibroblast cells. In addition, in vivo degradation of the hydrogel systems was examined to further consolidate the suitable use of GO.

## 2. Results and Discussion

### 2.1. Characterization of GO

**Figure 1:**
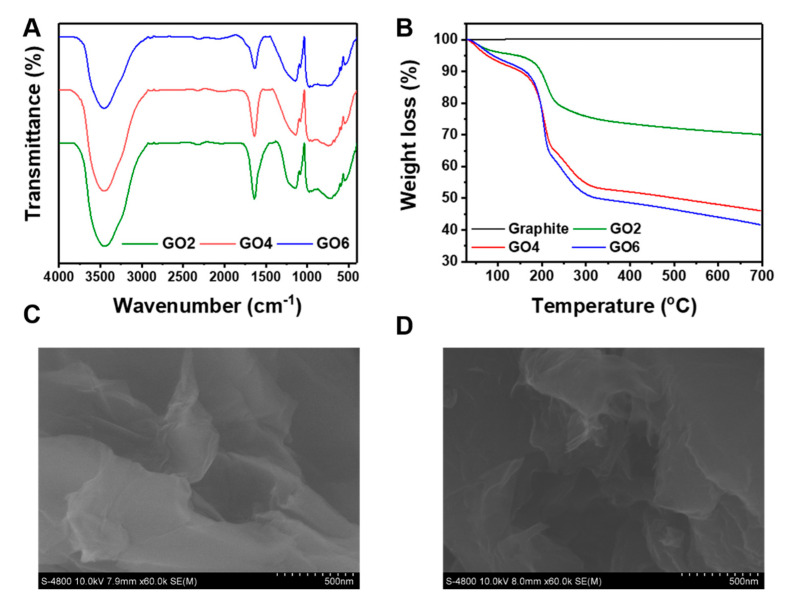
(**A**) Fourier-transform infrared (FTIR) spectra and (**B**) Thermogravimetric analysis of GO2 (red line), GO4 (blue line), GO6 (black line) and graphite (square dotted line); (**C**,**D**) Scanning electron microscopic (SEM) images of GO6 dispersed in deionized water solution.

Thanks to π-conjugating carbon-based network, graphite as well as graphene hardly exhibit the difference in the state of charges within their chemical structure, leading to a minimal dipole moment [35]. However, pristine graphite powder undertaken the oxidative process exposed various vibration signals, corresponding to the introduction of oxygenated functional groups on the basal plane of graphene, which indicates the successful treatment. In general, all FT-IR spectra in Figure 1A reveal the similar pattern of vibrations. It is clear that all GO samples were effectively fabricated by the modified Hummer method. In which, the strong trough at approximately 3455 cm^–1^ are contributed to the O-H stretching containing in hydroxyl and carboxylic acid. The nominal signals at under 3000 cm^–1^ are supposed to be the stretch mode of the sp^2^ and sp^3^ hybridizations of carbon skeletal network (C–C, C=C). The sharp bands at 1638 cm^–1^ correlate to the stretching vibration of the carbonyl group (C=O). The overlapped troughs at the wavenumbers of 1140 and 1082 cm^–1^ also indicate the C–O–C and C–CO–C stretching signals, containing in carboxyl and epoxy groups. It is widely evident that GO is functionalized by plentiful oxygen-enriched groups, such as carboxyl, hydroxyl, carbonyl and epoxy groups, as presented in our spectra [36,37,38].

Figure 1B shows the TGA plot of GO2, GO4, GO6 compared to graphite powder. It can be seen that, graphite specimen remains great stability and endurance under high temperature, a bit of weight gaining can be explained by the adsorption of nitrogen gas onto its surface. Notwithstanding the great stability of graphite, GO exhibits the low maintenance under the effect of temperature. The novel volatile functionalities introduced to graphene network have eased the decomposition of GO, resulting in the releases of CO_2_, H_2_O and other small molecules, which contributed to the mass losses [39,40]. Relatively, the weight loss of GO6 is two-fold higher than that of GO2, whereas the absorbance of the band at 3455 cm^–1^ of GO6 is just over 2.5 times higher than that of GO2. For this reason, it could be considered that the higher level of oxidative agent leads to the more oxygenated groups functionalized in GO specimen.

As mentioned, SEM analysis was used as a method to observe the morphology of GO. The analyzed sample was be prepared by dispersing GO6 powder into deionized water, of which dropped on aluminum foil to randomly take SEM images. Figure 1C reveals that the GO flakes expose a folded and thin-layered shape at a monoatomic thickness, which approves the successful exfoliation process in GO synthesis. Furthermore, those layers are likely to closely associate with each other due to their electrostatic interactions via the surficial functionalities, thereby expressing an aggregated form comprised of individual sheets and also space apart in some places (Figure 1D).

**Figure 2:**
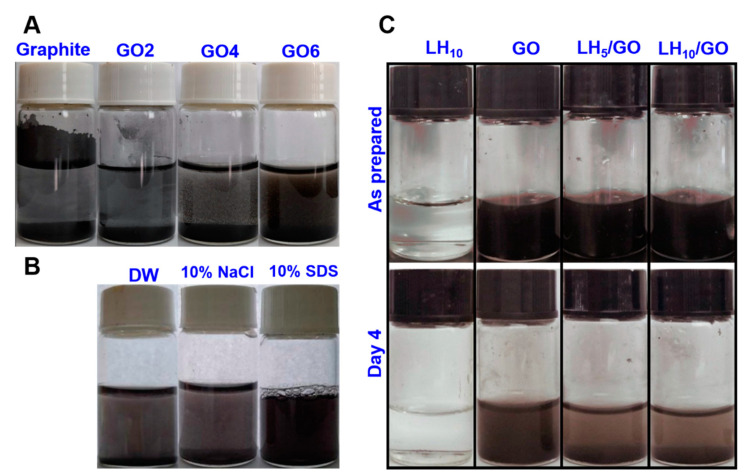
(**A**) Solubility of graphite and GOs after 12 h dispersed in deionized water. (**B**) Solubility of GO6 after 12 h dispersed in surfactant and salt. (**C**) Investigation of ion complexes formulation of graphene oxide and lidocaine hydrochloride.

Solubility of GOs is a prerequisite for this research. Therefore, the suspension of GOs in deionized water was evaluated to select the most suitable sample. According to Figure 2A, the graphite sample shows that there is black scum covered on the surface of the suspension, which is also less common in GO2 and GO4 samples. It is explained that the inherent hydrophobicity of graphene plates which dominants the amphiphilicity of GO2 and GO4, driving them high surface tension to diffuse in aqueous medium. On the other hand, the GO6 sample exhibits a good dispersibility after 12 h, that could be considered as the most appropriate sample for upcoming experiments. More importantly, presence of surfactants and NaCl in the dispersion medium not affected the dispersibility of GO6 (Figure 2B).

Figure 2C affirms the interaction of LH and GO by means of ionic complexation. It is clear that the LH_10_ solution remains apparent throughout the experiment. As just mentioned, the GO (LH_0_/GO) solution still reveals the well-dispersed property by day 4. Meanwhile, the LH-containing solutions (LH_5_/GO, LH_10_/GO) are less turbid than LH-free solution (LH_0_/GO) and show a significant aggregation on day 4. The phenomenon showed that there was a surface charge transition of GO from very negative to neutral. The neutralization, which mostly causes by the complexation between the basic group of LH and the acidic carboxyl of GO, induces GO plates to cluster together into a mass and conglomerate at the bottom.

### 2.2. Characterization of SA and GO-SA Hydrogels

**Figure 3:**
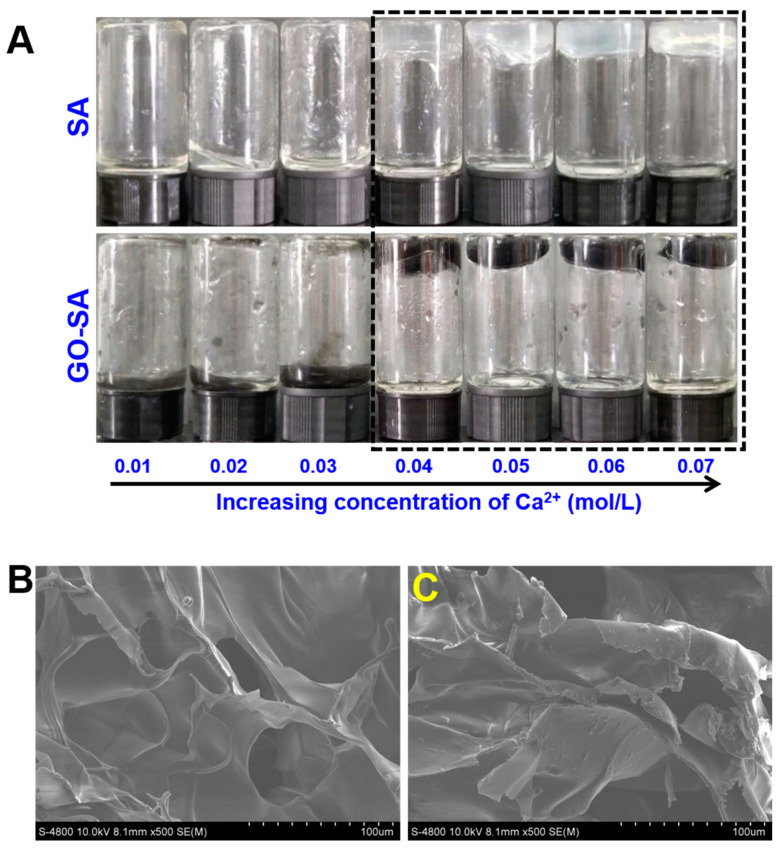
(**A**) Macroscopic images of hydrogel development. The experiment was conducted with blank SA hydrogel and GO-SA hydrogel. Both hydrogel systems commenced forming hydrogel at the feeding concentration of crosslinker of 0.04 mol/L, which zoning within a dotted area. SEM images of (**B**) SA and (**C**) GO-SA hydrogels.

Theoretically, in order to develop a hydrogel network, it is necessary to have an appropriate amount of crosslinking agent to determine the three-dimensional structure. Therefore, a series of CaCl_2_ solutions at multiple concentrations were used to examine gelation ability of this crosslinking agent. As a result, Table 1 indicates the gelation time of GO-free and GO-SA hydrogels in the presence of increasing concentration of Ca^2+^ ions. In particular, the concentrations below 0.4 mol/L cannot form hydrogel (Figure 3A). For higher amounts of crosslinker, the gelation time diminishes consistently with the increase of Ca^2+^ concentration, which was extensively reported in previous publications [41,42]. Moreover, the association of GO in the SA matrix also reveals short gelation time as compared to GO-free hydrogel. It could be explained that GO sheets offer synergetic effects to the GO-SA hydrogel formation by promoting diverse types of interactions: hydrogen bonds between oxygen-containing groups of GO plates and SA chains, divalent cation interactions of Ca^2+^ ions with GO plates and conventional divalent cation interaction generating hydrogel structure [43]. However, clinical practice shows that the gelation time at the crosslinker concentration of 0.06 mol/L is favorable to form hydrogel inside living body. Hence, this formulation was chosen to utilize in the in vivo experiment.

SEM analysis was also used to provide intuitive information in terms of interfacial morphology and crosslinking interaction of composite systems. As shown in Figure 3B, the pristine calcium alginate reveals a smooth surface, while Figure 3C is saliently witnessed convex and banded texture exposing on the surface of calcium alginate network. The embedded GO might be attributed to the distinction in morphological properties. Both composite matrices show porous architecture. However, the inner structure of GO-SA hydrogel is relatively denser than that of pure SA hydrogel since the aforementioned reasons, which contemplates the augmentation in tensile strength and stability of the hydrogel.

### 2.3. Degradation of SA and GO-SA Hydrogels

**Figure 4:**
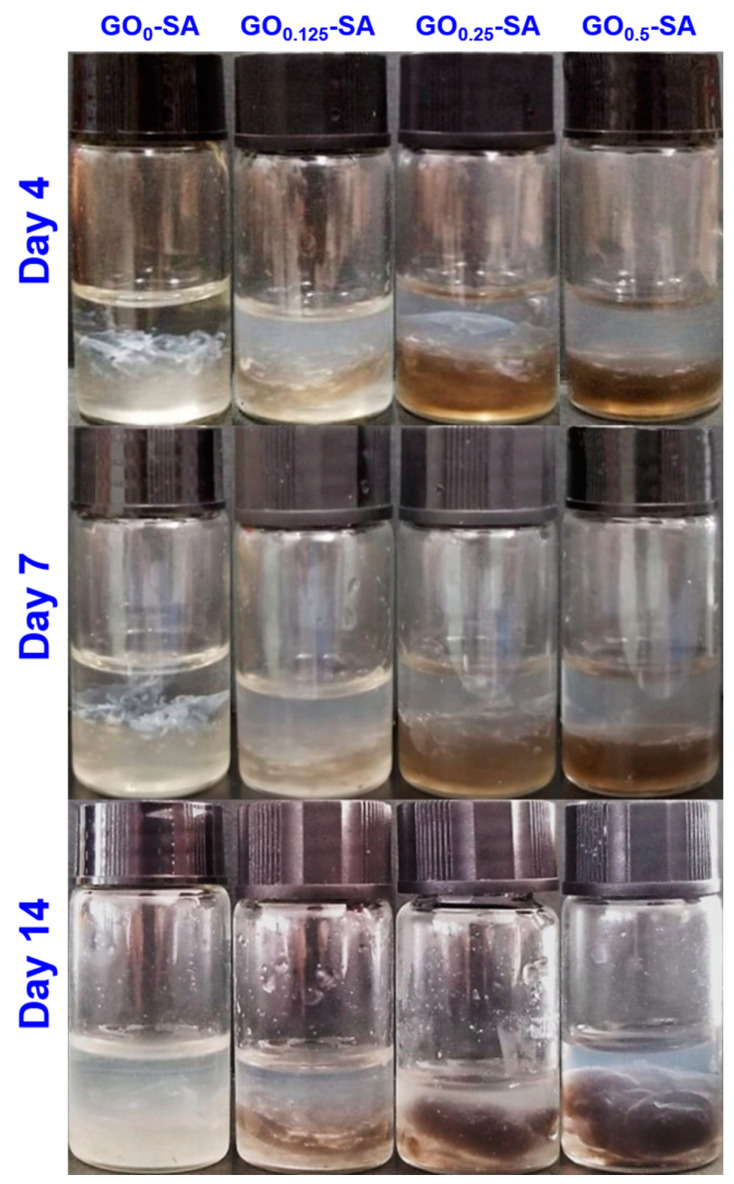
Degradation of SA and GO-SA hydrogels in PBS.

By observing the macroscopic morphology of hydrogel samples in PBS medium (Figure 4), it is recognized that the GO_0_/SA present rapid swelling at the interfacial surface. After 2 weeks, the hydrogel mostly decayed into an opaque liquid which contains decomposed SA polymer chains. This quick decay might be contributed by the ion exchange between divalent ions (Ca^2+^) in calcium alginate and monovalent ions existing in PBS solution [44]. For GO-SA hydrogels, there is a more stable tendency in the aqueous medium. Indeed, the higher concentration of GO boosts the synergetic effects of GO on the reinforcement of hydrogel, thereby lessening the degradation of the hydrogel systems under fluid condition [43,45].

### 2.4. In Vitro Drug Release

**Figure 5:**
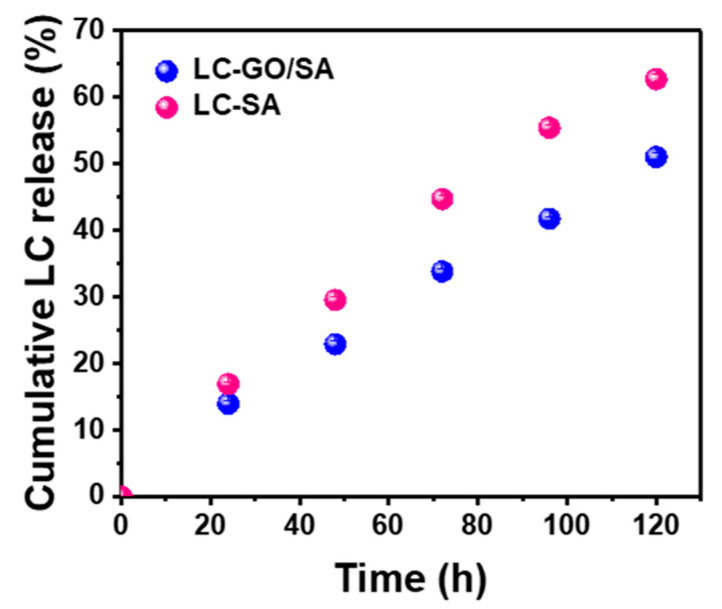
The in vitro lidocaine hydrochloride release profiles of SA and GO-SA systems in PBS solution. The error bars in the graph represent standard deviations (n = 4).

SA and GO-SA hydrogels were used to encapsulate the model anesthetic (LH), in which the soluble form of lidocaine, lidocaine hydrochloride, was released in PBS medium with time. The behavior of release profiles, which illustrates in Figure 5, is considered to be relatively linear and stable without burst release. The higher slope in the release of SA hydrogel reveals the faster drug release rate compared with that of GO-SA hydrogel. After 120 h, 62.73 ± 0.70% encapsulated drug had diffused out of SA hydrogel network into external medium, whereas there was only 51.10 ± 0.44% LH had been released from GO-SA hydrogel. It is persuasive that our facile design of graphene oxide-incorporated polymeric hydrogel is efficient to delivery and control sustaining release of lidocaine for local anesthesia.

### 2.5. In Vitro Biocompatibility of Hydrogels

**Figure 6:**
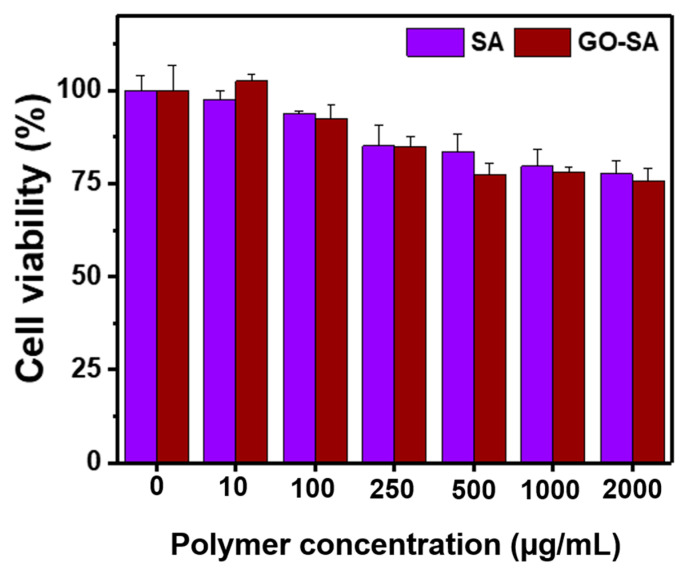
The in vitro cell viability of L929 mouse fibroblast cells exposed with different concentration of SA and GO-SA hydrogels. The error bars in the graph represent standard deviations (n = 4).

Non-toxic property of hydrogels or hydrogelators is an essential factor for biomaterials in in vivo applications. In order to prove biocompatibility of hydrogels, various concentrations of SA or GO-SA hydrogels were co-incubated with L929 mouse fibroblast cells for 48 h followed by evaluating the viability using MTT assay. The cell viability of L929 mouse fibroblast cells co-cultured with hydrogels is shown in Figure 6. As expected, cell viability of fibroblast cells was high (>80%) even they were exposed with 2000 µg/mL of hydrogel precursors, which implied the biocompatibility of GO-SA hydrogels.

### 2.6. In Vivo Hydrogel Formation

**Figure 7:**
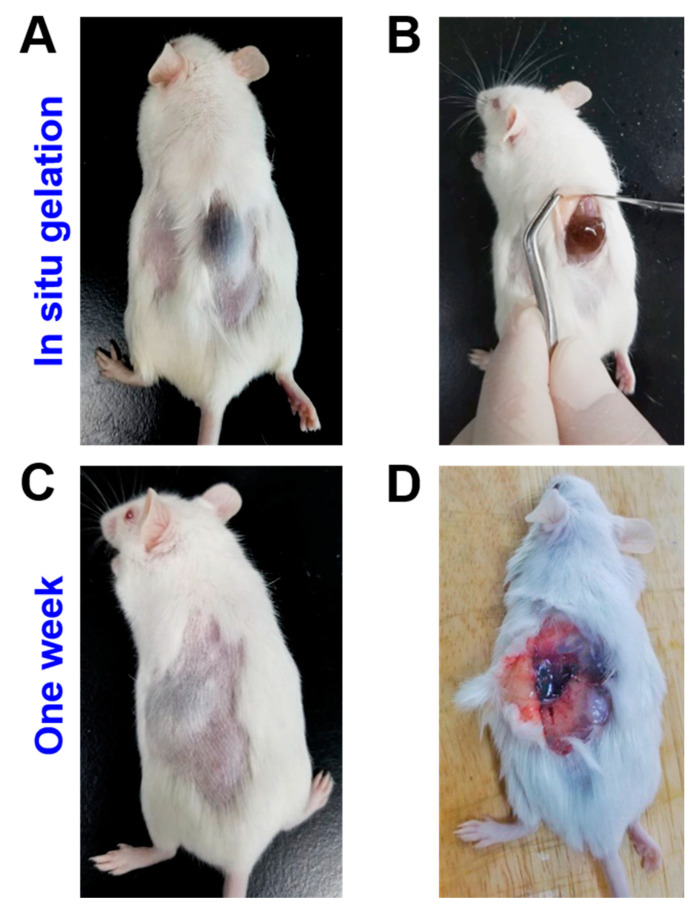
Photographs of the in vivo study: (**A**) Grey bumps of GO-SA hydrogel cured under the mice’s skin after hypodermic injection, (**B**) Incision to test stable gel formation of the hydrogel. (**C**) After a week, the hydrogel remained stable without inflammation and (**D**) After 1 week, disintegration of the GO-SA hydrogels was observed. This is mainly due to the slow exchange of cations and salt effects.

The in vivo study has fully confirmed the utility of the GO-SA hydrogel system, as shown in Figure 7. After subcutaneous administration, the gel has been successfully implanted at specific site of mice, which demonstrates an appropriate gelation time to be used as an injectable hydrogel system. After injection, the mice have normal function and no abnormal behavior within 1 week. When dissected, the hydrogel showed sign of degradation, while internal organs surrounding the gels were not infected or inflamed. It is clear that the GO-integrated SA hydrogel can be used as a local anesthetic delivery system.

In the GO-SA hydrogel system, the in vivo formation mainly underlies the physical-crosslinking of Ca^2+^ ions and a pair of buckled α-L-guluronic acid (G) residues. This combination induced a rapid gel formation. Furthermore, with the use of GO, there could be other kinds of physical crosslinks, such as hydrogen bonding between oxygen-containing groups of GO plates and SA chains and divalent cation interactions of Ca^2+^ ions with GO plates, appearing in the hydrogel.

## 3. Conclusions

Our study aims to design a facile hydrogel system from SA, and use synthetic GO for controlled release of lidocaine. Herein, the GO is successfully fabricated by the modified Hummer method without extra oxidative agents. The chemical structure and oxidation level of GO are confirmed by FT-IR and TGA. GO6 with the highest oxidation level reveals good solubility and dispersity in aqueous medium. Furthermore, the ionic complexation of GO and LH constitutes a key factor to enhance the loading capacity and controlled release for the hydrogel system. The result showed that the blending of 4% SA solution and 0.06 mol/L CaCl_2_ solution gives the most appropriate result due to the suitable gelation time. The incorporation of 0.5% GO in SA matrix shows the quicker gelation time as well as good stability and release behavior of LH. Furthermore, the in vivo study on mice also yields a positive result with high compatibility and degradation, which shows that the LH-loaded GO-SA hydrogel can be a successful drug delivery system for controlled release.

## 4. Materials and Methods

### 4.1. Materials

Graphite powder, concentrated sulfuric acid (H_2_SO_4_, 95–98%), and potassium permanganate (KMnO_4_, 99%) were obtained from Sigma-Aldrich (St. Louis, MO, USA). Alginic acid sodium salt (SA), phosphate buffered saline pH 7.4 (PBS) and anhydrous calcium chloride (CaCl_2_, 98%) was provided by Merck (Darmstadt, Germany). Hydrogen peroxide (H_2_O_2_, ~30%), concentrated hydrochloride (HCl, 37%) and lidocaine hydrochloride monohydrate (LH, 98%) were purchased from Fisher Chemicals (Trenton, NJ, USA).

### 4.2. Methods

Morphological properties of GO and hydrogels were observed by scanning electron microscope (SEM, JSM-IT100, Tokyo, Japan). The introduction of oxygen-containing groups of GO was detected by Fourier-transformed infrared spectrophotometer (FT-IR, PerkinElmer, Waltham, MA, USA) using KBr pellet method. Thermogravimetric analysis (TGA, Mettler Toledo TGA/DSC 3+, Sweden) was carried out to investigate thermal stability of GO, and to preliminarily examine the oxidative level the samples. The TGA plot was recorded from 30 to 700 °C at the ramping rate of 10 °C·min^–1^ under the continuous flow of N_2_ gas. All resultant data were illustrated as mean ± standard deviations.

### 4.3. Syntheses of GOs

The synthesis of GO in this study was attempted on the basis of Hummers method with minimal modification [46,47]. Our approach is considered to be NaNO_3_-free methods, advancing the low consumption of reagent but remaining the high yield, in which we utilized sulfuric acid as an intercalator and acidic oxidizing medium, KMnO_4_ as a strong oxidative agent, water and hydrogen peroxide as exfoliators [48,49]. In brief, three suspensions of 0.8 g graphite powder in 15 mL of concentrated H_2_SO_4_ were prepared, stirring at 0 °C for 1 h. Particular levels of oxidative agent (2, 4, 6 g) were gradually dissolved into the solutions, the reactions were carried out for 3 h. Based on the oxidative agents the samples were denoted as GO2, GO4, GO6. Sequentially, the mixture was placed at 35 °C, stirring for 2 h. In order to gain separate GO lattices, 60 mL of deionized water (DIW) and 6 mL of H_2_O_2_ (30%) were, respectively, added and remained the same conditions for 1 h. Ultimately, 150 mL DIW was used to propagate the syntheses of GOs. The resultant mixtures were rinsed by HCl 5% solution and centrifuged in the first place. Then precipitates were rinsed again with distilled water and centrifuged until the pH reached neutral. The precipitates were lyophilized for further use.

### 4.4. Preparation of GO and LH Ionic Complexes

After the preparation of GOs, dispersibility test is convincing to select the most suitable GO for the drug delivery system. This kind of GO must attain a high solubility and sustaining suspension in aqueous medium. Three samples of GOs were dissolved in distilled water and put in an ultrasonic bath for 30 min. Those well-dispersed samples were all placed stable conditions for 12 h and observed. As hypothesized, the ionic complexes between GO and LH might be developed due to the opposite charges, of which interaction might generate a significant change in electric charge of multivalent GO. Therefore, we demonstrated it by preparing and observing four dispersions that had the final concentrations of GO at 0.25 mg/mL and LH at 0, 5, 10 mg/mL, which were expressed as LH_0_/GO, LH_5_/GO, LH_10_/GO, respectively, and a solution of LH (10 mg/mL), which was expressed as LH_10_.

### 4.5. Fabrication of SA and GO-SA Hydrogels

The sodium alginate hydrogel systems could be formulated by versatile crosslinking agents, either chemical or physical [50,51]. In order to simplify the fabrication, we decided to elect calcium ion (Ca^2+^) as a physical crosslinker using the CaCl_2_ source. In this experiment, GO-free SA and GO-SA hydrogels formation were prepared by a series of CaCl_2_ concentrations, as designated by SA and GO-SA hydrogels, respectively. GO-SA suspension was prepared by mixing 8% SA solution with 0.5% GO6 dispersion in DIW (*w*/*v*). Then, both mixtures were sonicated in an ultrasonic bath for 30 min to yield the SA and GO-SA solutions. A set of seven aqueous solutions of CaCl_2_ was prepared by diluting from the concentration of 0.07 to 0.01 mol/L at intervals of 0.01 mol/L. In order to fabricate the hydrogels, 1 mL crosslinker solution at various concentrations was blended with 1 mL polymer solution, the mixture was gently shaken to be well-mixed and contemporaneously record gelation time of each combination using a stopwatch.

### 4.6. Degradation Study of SA and GO-SA Hydrogels

Notwithstanding universal applications of pure alginate hydrogel in biomedical field, the lack of mechanical strength and inevitable swelling in solution have exposed as remarkable limits in some specific approaches [52]. In addition to the potential in improved adsorption capacity, GO was widely known that can effectively integrate with polymeric matrix, particularly alginate, to reinforce the hydrogel network and complement the structural and mechanical properties of it [53,54]. In order to evaluate the stability of SA and GO-SA hydrogels, the degradation experiment was utilized. Briefly, GO6 (0.125%, 0.25%, 0.5% *w*/*v*), SA (8% *w*/*v*), LH (2% *w*/*v*) and CaCl_2_ (0.06 mol/L) stock solutions were prepared with deionized water (Table 2). Equivalent volume of above solutions was mixed to develop hydrogels, and those specimens was compared with a GO-free hydrogel. After complete gelation, the specimens were placed in 3 mL of PBS solution to further observe.

### 4.7. Loading and In Vitro Release of LH

In order to testify the controlled release effect of GO towards SA hydrogel system, the model analgesic drug (lidocaine hydrochloride) was loaded into the GO-SA hydrogel and blank hydrogel (without GO). Before complete blending of individual component solutions, the 2% LH solution (*w*/*v*) and 0.5% GO6 solution (*w*/*v*) were mixed at the proportionate ratio and stirred for 1 h. Gelation of GO-SA hydrogel was conducted as previously aforementioned, in which the drug solution, SA (8% *w*/*v*) and CaCl_2_ (0.06 mol/L) solutions were combined at the ratio of 2:1:1, respectively. For the GO-free SA hydrogel, the GO6 solution was replaced by deionized water. After accomplishing gelation, the hydrogels were slightly immersed in 3 mL PBS solution and placed at body temperature (37 °C). At regular time intervals of a day, 1.5 mL release solution of sample aliquots was pipetted out and stored for analysis. An equivalent volume of medium was refilled and kept maintain the previous condition. Eventually, the amount of LH in the collected sample aliquots was measured by UV-Vis spectrophotometer at the wavelength of 265 nm, whereas PBS was utilized as the reference standards. This experiment was carried out in triplicate.

### 4.8. In Vitro Cytotoxicity

The L929 cells were purchased from Korean Cell Line Bank (KCLB) and cultured in a DMEM supplemented with 10% fetal bovine serum and 1% penicillin-streptomycin (1%, (*w*/*v*)). Subsequently, the cell flasks were incubated at 37 °C in a humidified 5% CO_2_˗95% air atmosphere. To confirm the biocompatibility, the cells were cultured with various concentrations of copolymers. After 48 h, 20 µL MTT solution (from 5 mg/mL stock solution) was added and incubated for 3 h at 37 °C. At the same time, the purple crystals were dissolved in DMSO and the viability was examined with Microplate reader by measuring the absorbance at 490 nm. The L929 cells cultured with only fresh culture medium were used as control.

### 4.9. In Vivo Hydrogel Formation and Compatibility

This study aims to assess the in vivo hydrogel formation and compatibility of hydrogel system on animal model. The animal experiments performed in this work were approved by the Institutional Committees of Jiaxing University and the animal experiments were conducted according to the guidelines of Jiaxing University. Mice (BALB/c mice, 20.0 ± 2.0 g) were used to carry out the experiment. The mice were anesthetized by introducing 5% of isoflurane in oxygen via a vaporizer. Separate blends of GO-SA mixture including LH (0.5% *w*/*v*) and crosslinker solution were prepared. Then, the blends of GO/SA and CaCl_2_ were placed in the dual syringe before the injection [55,56]. Then the mixture was injected subcutaneously. After 30 min, a mouse was sacrificed for gel observation. The status of hydrogel formation was observed to demonstrate injectable capability. After 1 week, the mice were also sacrificed and the compatibility of the hydrogel system was assessed.

## Figures and Tables

**Table 1:** Gelation time of GO-free and GO-integrated SA hydrogels. Gelation time recorded in seconds.

	Ca^2+^ Concentration (mol/L)						
	0.01	0.02	0.03	0.04	0.05	0.06	0.07
**SA gel (s)**	No gel formation			71.7 ± 1.5	65.3 ± 1.2	49.0 ± 1.0	32.7 ± 2.1
**GO-SA gel (s)**	No gel formation			61.7 ± 0.6	52.3 ± 0.6	41.7 ± 1.2	30.3 ± 1.5

**Table 2:** The compositions of hydrogel samples before testing aqueous degradation.

Sample No	GO6 (mL)	SA 8% (mL)	LH 2% (mL)	Ca^2+^ (0.06M) (mL)	PBS (mL)
**GO_0_-SA**	0.5 (0%)	0.5	0.5	0.5	3.0
**GO_0.125_-SA**	0.5 (0.125%)	0.5	0.5	0.5	3.0
**GO_0.250_-SA**	0.5 (0.250%)	0.5	0.5	0.5	3.0
**GO_0.500_-SA**	0.5 (0.500%)	0.5	0.5	0.5	3.0

## Data Availability

The data presented in this study are available on request from the corresponding author.
